# Supplementary material for: The causal role of circulating inflammatory markers in osteoporosis: a bidirectional Mendelian randomized study
Source: Front Immunol. 2024 Jul 18;15:1412298. doi: 10.3389/fimmu.2024.1412298 (PMC11291241; doi:10.3389/fimmu.2024.1412298)
Supplement: Supplementary file 1 [file DataSheet_1.zip › supplementary material/OSTEOPOROSIS-CIM/GCST90274829.tsv.gz_forest.pdf]

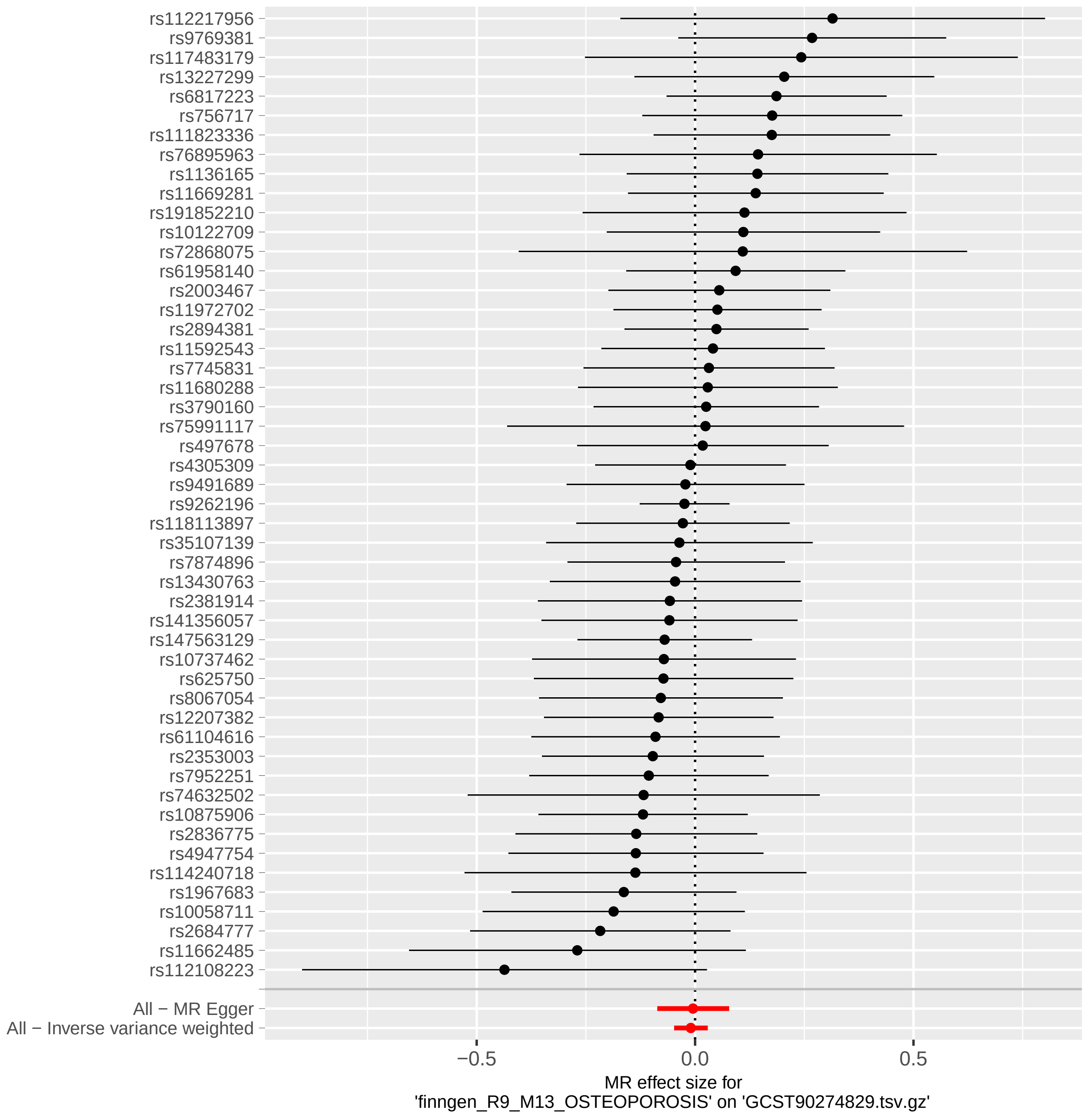
rs112217956
rs9769381
rs117483179
rs13227299
rs6817223
rs756717
rs111823336
rs76895963
rs1136165
rs11669281
rs191852210
rs10122709
rs72868075
rs61958140
rs2003467
rs11972702
rs2894381
rs11592543
rs7745831
rs11680288
rs3790160
rs75991117
rs497678
rs4305309
rs9491689
rs9262196
rs118113897
rs35107139
rs7874896
rs13430763
rs2381914
rs141356057
rs147563129
rs10737462
rs625750
rs8067054
rs12207382
rs61104616
rs2353003
rs7952251
rs74632502
rs10875906
rs2836775
rs4947754
rs114240718
rs1967683
rs10058711
rs2684777
rs11662485
rs112108223
All − MR Egger
All − Inverse variance weighted
−0.5
0.0
0.5
MR effect size for
'finngen_R9_M13_OSTEOPOROSIS' on 'GCST90274829.tsv.gz'
